# Involvement of the Bradykinin B_1_ Receptor in Microglial Activation: *In Vitro* and *In Vivo* Studies

**DOI:** 10.3389/fendo.2017.00082

**Published:** 2017-04-19

**Authors:** Keren Asraf, Nofar Torika, Abraham Danon, Sigal Fleisher-Berkovich

**Affiliations:** ^1^Department of Clinical Biochemistry and Pharmacology, Ben-Gurion University of the Negev, Beer-Sheva, Israel

**Keywords:** bradykinin, brain inflammation, HOE 140, microglia, R-715

## Abstract

The importance of brain inflammation to Alzheimer’s disease (AD) pathogenesis has been accepted of late, with it currently being held that brain inflammation aggravates AD pathology. One important aspect of brain inflammation is the recruitment and activation of microglia, a process termed microgliosis. Kinins and bradykinin (BK), in particular, are major pro-inflammatory mediators in the periphery, although all of the factors comprising the kinin system have also been described in the brain. Moreover, it was shown that the amyloid β (Aβ) peptide (a component of AD plaques) enhances kinin secretion and activates BK receptors that can, in turn, stimulate Aβ production. Still, the role of bradykinin in modulating brain inflammation and AD is not completely understood. In this study, we aimed to investigate the roles of the bradykinin B_1_ receptor (B_1_R) and bradykinin B_2_ receptor (B_2_R) in regulating microglial secretion of pro-inflammatory factors *in vitro*. Furthermore, the effects of intranasal administration of specific B_1_R and B_2_R antagonists on Aβ burden and microglial accumulation in the brains of transgenic AD mice were studied. The data obtained show that neither R-715 (a B_1_R antagonist) nor HOE 140 (a B_2_R antagonist) altered microglial cell viability. However, R-715, but not HOE 140, markedly increased lipopolysaccharide-induced nitric oxide (NO) and tumor necrosis factor-alpha (TNF-α) release, as well as inducible nitric oxide synthase expression in BV2 microglial cells. Neither antagonist altered NO nor TNF-α production in non-stimulated cells. We also showed that intranasal administration of R-715 but not HOE 140 to 8-week-old 5X familial AD mice enhanced amyloid burden and microglia/macrophage accumulation in the cortex. To conclude, we provide evidence supporting a role of B_1_R in brain inflammation and in the regulation of amyloid deposition in AD mice, possibly with microglial/macrophage involvement. Further studies are required to test whether modulation of this receptor can serve as a novel therapeutic strategy for AD.

## Introduction

Alzheimer’s disease (AD) is a prevalent neurodegenerative disease (1) that is characterized by two neuropathological hallmarks, namely the deposition of amyloid β plaques and the accumulation of neurofibrillary tangles (2, 3). The role that brain inflammation plays in AD pathogenesis has only recently been appreciated. Currently, brain inflammation is thought to contribute to and to exacerbate AD pathology (4, 5). One important aspect of the central immune response to brain inflammation is microglial activation.

Microglia are the resident phagocytes of the brain. These cells use their processes to scan the brain for pathogens and debris (6). Microglia also maintain brain plasticity and remodel synapses (7, 8). In AD, microglia bind soluble amyloid β (Aβ) oligomers and fibrils and become activated. The activated microglia then start to engulf Aβ fibrils by phagocytosis. Inefficient phagocytosis of Aβ has been identified as a major pathogenic pathway (5). Microglia are likely to exist in a range of phenotypic states during chronic inflammation in AD. Upon binding to Aβ, microglia release pro-inflammatory molecules, such as interleukin-1 β (IL-1β), tumor necrosis factor-alpha (TNF-α), and IL-12, as well as reactive oxygen species, like nitric oxide (NO) (9–11). In turn, pro-inflammatory conditions promote neuronal damage mediated by Aβ and decrease the phagocytosis of these oligomers and their degradation by microglia (12, 13).

Although much is known of the molecular basis of initiating signals and pro-inflammatory chemical mediators in brain inflammation, it has only recently become apparent that endogenous stop signals are critical players at early checkpoints during the various stages of brain inflammation. Some neuropeptides that are produced during the ongoing inflammatory response have emerged as endogenous anti-inflammatory agents that participate in the regulation of processes that ensure self-tolerance and/or inflammation resolution. Neuropeptides, such as kinins, can regulate brain inflammation and affect microglial functions both *in vitro* and *in vivo* (14, 15). Thus, the release of these factors can determine whether microglia assume a neuroprotective phenotype.

Kinins, in particular bradykinin (BK), are pro-inflammatory mediators in the periphery. At peripheral sites, BK can elicit all of the major signs of inflammation, namely pain, hyper-perfusion, and increased vascular permeability (16–19). All kinin system components have also been described in the central nervous system (20). Indeed, high BK levels are found after brain trauma and ischemia (21). Furthermore, it was shown that Aβ upregulates BK receptors and kinin release, followed by BK-induced Aβ synthesis (22). Still, the role that bradykinin plays in AD modulation is not completely understood. BK activates two types of receptors, namely, the B_1_ receptor [bradykinin B_1_ receptor (B_1_R)] and the B_2_ receptor [bradykinin B_2_ receptor (B_2_R)] (23, 24). B_2_R is a constitutive receptor and has high affinity for BK, while B_1_R is generally upregulated following tissue injury and binds with high affinity to des-Arg9-BK, a kinin metabolite (24). In the brain, microglial cells express both receptors (14, 25).

In the present study, our intent was to investigate the contributions of B_1_R and B_2_R in mediating microglial inflammation *in vitro*. Moreover, the *in vivo* influence of intranasal administration of specific B_1_R and B_2_R antagonists on Aβ burden and microglial accumulation in brains of transgenic AD mice was considered.

## Materials and Methods

### Cell Cultures

The BV2 microglial cell line (provided by Prof. Rosario Donato, Department of Experimental Medicine and Biochemical Sciences, University of Perugia) was seeded in 6-well, 24-well, or 96-well plates at densities of 1 × 10^6^, 3 × 10^5^, and 2 × 10^4^ cells per well, respectively. Cells were maintained in RPMI-1640 supplemented with 10% fetal calf serum and 0.4 mM l-glutamine. To create a sterile environment, 100 U/ml of penicillin and 100 µg/ml of streptomycin were added. Cells were grown in humidified atmosphere of 5% CO_2_ at 37°C. At the beginning of each experiment, the cells were incubated with serum-free medium (SFM) for 4 h, followed by a 22-h incubation with the indicated test agents in SFM supplemented with 0.1% bovine serum albumin (BSA) and 10 mM HEPES (pH 7.4). BV2 cells were treated with R-715, a B_1_R selective antagonist, and HOE 140, a B_2_R selective antagonist, both purchased from GL Biochem (Shanghai, China), lipopolysaccharide (LPS) from *Escherichia coli* serotype 055:B5 was purchased from Sigma Aldrich (St. Louis, MO, USA).

### Cell Count

At the end of each experiment, cells were harvested after incubation with 1 ml SFM for 1 h at 4°C and counted using a Z1 Coulter counter (Coulter Electronics, Miami, FL, USA).

### Cell Viability

Cell viability was determined by a Cell Proliferation Kit (XTT) (Biological Industries, Kibbutz Beit-Haemek, Israel) according to the manufacturer’s instructions. The assay was performed using a microplate reader (Bio-Rad model 680).

### Determination of NO Levels (Griess Reaction)

Nitrite levels were determined in the culture supernatants using the Griess reaction. Nitrite standard curve samples or supernatants (100 µl each) were mixed with 100 µl Griess reagent (Sigma-Aldrich) in 96-well plates. Thereafter, the plates were incubated for 15 min in the dark at room temperature. Nitrite levels were measured with a microplate reader at 540 nm.

### Determination of TNF-α Levels (ELISA)

Tumor necrosis factor-alpha levels were measured using an enzyme-linked immunosorbent assay (ELISA) kit (BD Biosciences, San Diego, CA, USA) according to the manufacturer’s instructions.

### SDS Polyacrylamide Gel Electrophoresis and Western Blot Analysis

The expression levels of inducible nitric oxide synthase (iNOS) protein in BV2 microglial cells were analyzed by Western blot (26). Briefly, cells were harvested using lysis buffer (20 mM HEPES pH 7.4, 150 mM NaCl, 1 mM EDTA, 1 mM EGTA, 10% glycerol, 1 mM MgCl_2_, 1% Triton X-100, and 1% deoxycholic acid) containing a protease inhibitor cocktail. Cells lysates were incubated at 4°C for 30 min, followed by a 15 min centrifugation (12,000 *g*) at 4°C. Thereafter, protein levels were determined by Bradford assay (Bio-Rad). Aliquots of whole cell lysates containing 40 µg protein were denatured and separated on 7.5% polya-crylamide-SDS gels and transferred to nitrocellulose membranes. Non-specific sites were blocked by 4% BSA (90 min incubation at room temperature). This was followed by overnight incubation at 4°C with rabbit anti-iNOS antibodies (1:500; Cayman Chemicals, Ann Arbor, MI, USA). After washing, the membranes were incubated with IgG-horseradish peroxidase (HRP)-conjugated donkey anti-rabbit antibodies (1:5,000; GE Healthcare, Buckinghamshire, UK) for 90 min at room temperature. Finally, enhanced chemiluminescence solution was added, and the membranes were exposed to X-ray film (Fuji medical X-ray film, FujiFilm). Protein levels were normalized to β-actin levels using mouse monoclonal anti-β-actin antibodies (1:25,000; MP Biological, Santa Ana, CA, USA) and HRP-conjugated goat anti-mouse antibodies (1:20,000; Jackson ImmunoResearch Laboratories, West Grove, PA, USA). A computerized image analysis system (EZ Quant-Gel 2.2, EZQuant Biology Software Solutions, Israel) was used for semi-quantitative analysis.

### Mice

Wild-type (WT) C57BL/6 mice were purchased from Harlan Israel (Jerusalem, Israel). Transgenic 5X familial AD (5XFAD) mice were provided by Prof. Robert Vassar (Department of Cell and Molecular Biology, Northwestern University). 5XFAD mice include five mutations under the transcriptional control of the neuron-specific mouse Thy-1 promoter, with three mutations in the human APP695 gene (Swedish K670N, M671L, Florida I716V and London V717I) and two mutations in the human presenilin-1 gene (M146L, L286V). At the age of 2 months, 5XFAD mice evolve Aβ accumulation and gliosis (27). The human APP gene was detected by PCR analysis of mice tail tissue DNA. Mice were placed in cages at temperatures of 22 ± 2°C and 65% humidity. Food and water supply was made available, and a 12 h light/dark cycle was maintained. All animal studies were performed according to the recommendations of Institutional Animal Care and Use Committee of Ben-Gurion University of the Negev. The protocol employed was approved by this committee (approval number IL-30-08-2011).

In the first experiment, 8-week-old mice were divided into three groups, namely WT mice treated with R-715, 5XFAD mice treated with saline, and 5XFAD mice treated with R-715. In the second experiment, mice were again divided into three groups, namely WT mice treated with HOE 140, 5XFAD mice treated with saline, and 5XFAD mice treated with HOE 140. The mice received daily intranasal treatment of 1 mg/kg per day for 18 days (5 days/week).

### Immunohistochemistry

Mice were anesthetized upon intra-peritoneal injection of 0.2 ml ketamine–xylazine mixer (1:1). Brains were removed following cold PBS cardiac perfusion and were divided into two hemispheres. One hemisphere from each mouse brain was incubated overnight in 4% paraformaldehyde solution at 4°C, followed by incubation in 30% sucrose solution at 4°C for 2 days. The hemispheres were then frozen in molds containing tissue adhesive (O.C.T. compound, Tissue-Tek, Torrance, CA, USA) at −80°C. All hemispheres were cut into sagittal sections (40 µm) using a cryostat and maintained at −20°C in PBS:ethyleneglycol:glycerol (2:1:1) non-freezing solution. Free-floating sections were washed using 0.05% PBS/Tween 20 and permeabilized using 0.5% PBS/Triton X-100. After blocking non-specific binding using antibody diluent solution (GBI Labs, Mukilteo, WA, USA), sections were incubated for 2 h at room temperature with rabbit anti-human Aβ (1:250; a gift from Prof. Alon Monsonego, The Shraga Segal Department of Microbiology and immunology, Faculty of Health Sciences and the National institute of Biotechnology in the Negev, Ben-Gurion University of the Negev) and rat anti-mouse/human CD11b antibodies (1:25, Biolegend). Primary antibodies were diluted in antibody diluent solution. Thereafter, the sections were rinsed and incubated for 1 h at room temperature with the appropriate secondary antibody, i.e., Cy^3^-conjugated donkey anti-rabbit IgG (1:1,000) or Alexa fluor 488-conjugated goat anti-rat IgG (1:250), both from Jackson ImmunoResearch Laboratories. Secondary antibodies were diluted in 0.05% PBS/Tween 20. After washes, the sections were mounted with mounting medium containing DAPI (Vector labs) on charged slides and stored at 4°C. An Olympus FluoView FV1000 confocal microscope (Olympus, Hamburg, Germany) at 1,024 × 1,024 pixel resolution with a ×10 objective was used for imaging. In each experiment, five sections from the cortex of each animal were analyzed. Aβ and CD11b staining was quantified using the ImageJ software version 1.40C (NIH). Average fluorescent Aβ- and CD11b-containing areas were calculated for each treated group.

### Statistical Analysis

For each experiment, results are presented as the mean ± SEM. To assess the statistical significance of differences between treatment groups, one-way analysis of variance was performed, followed by a *post hoc* multiple comparison test (Tukey–Kramer Multiple Comparison Test). *P* < 0.05 was considered statistically significant.

## Results

**Figure 1:**
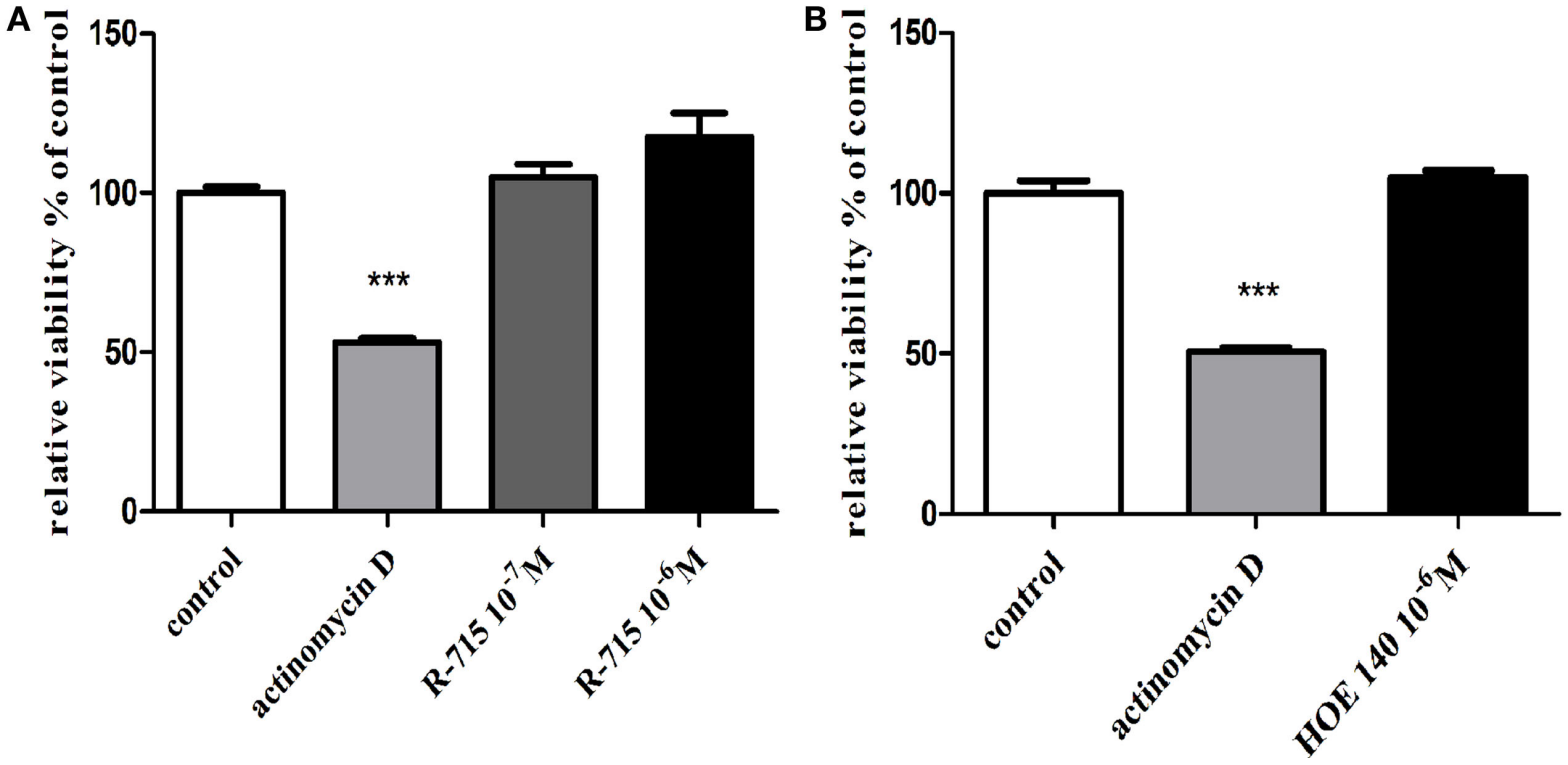
**Effects of bradykinin B_1_ receptor (B_1_R) and bradykinin B_2_ receptor (B_2_R) antagonists on the viability of BV2 cells**. Cells were incubated with **(A)** the B_1_R antagonist R-715 (10^−7^ and 10^−6^ M) or **(B)** the B_2_R antagonist HOE 140 (10^−6^ M) for 24 h. At the end of the experiment, viability was assessed by the XTT assay. Results are given as mean ± SEM, *n* = 4; a one-way analysis of variance and Tukey–Kramer Multiple Comparison Test were performed to determine statistical significance; ***P < 0.001 versus the control.

Serving as a positive control, actinomycin D, significantly reduced BV2 microglial cell viability (Figure 1). By contrast, neither the B_1_R antagonist R-715 at concentrations of 10^−7^ and 10^−6^ M (Figure 1A) nor the B_2_R antagonist HOE 140 at a concentration of 10^−6^ M (Figure 1B) altered microglial cell viability, as measured with XTT assay.

**Figure 2:**
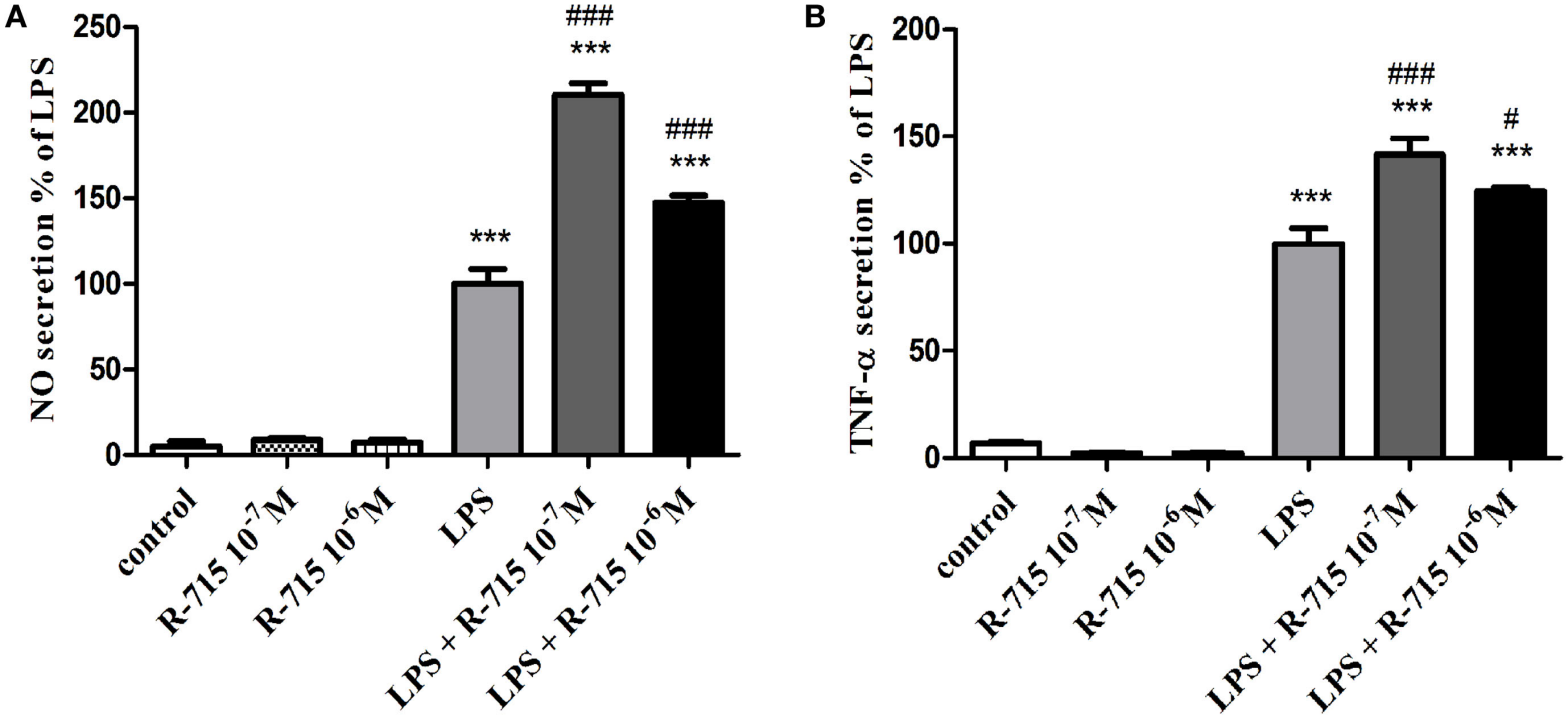
**Effects of the bradykinin B_1_ receptor (B_1_R) antagonist on basal and lipopolysaccharide (LPS)-induced NO and tumor necrosis factor-alpha (TNF-α) release from BV2 cells**. Cells were incubated with the B_1_R antagonist R-715 (10^−7^ and 10^−6^ M) in the presence or absence of LPS (7 ng/ml) for 24 h. NO **(A)** and TNF-α **(B)** levels were measured in the media, and the cells were counted. Results are representatives of three independent experiments and are presented as mean ± SEM, *n* = 3–6; statistical significance was assessed by one-way analysis of variance followed by a Tukey–Kramer Multiple Comparison Test; ***P < 0.001 versus the control, ^###^*P* < 0.001 versus LPS, and ^#^*P* < 0.05 versus LPS.

**Figure 3:**
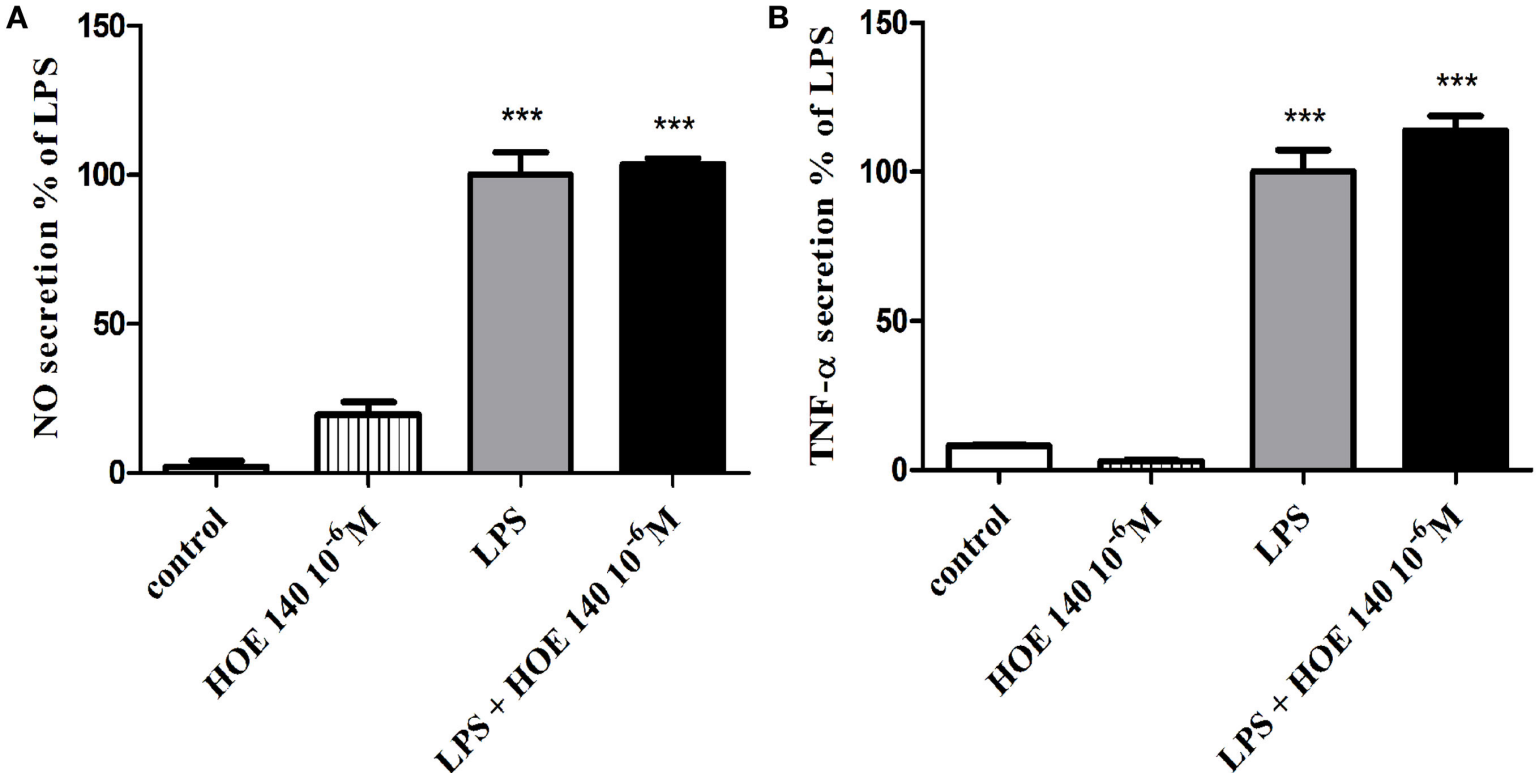
**Effect of the bradykinin B_2_ receptor (B_2_R) antagonist on basal and lipopolysaccharide (LPS)-induced NO and tumor necrosis factor-alpha (TNF-α) release from BV2 cells**. Cells were incubated with the B_2_R antagonist HOE 140 (10^−6^ M) in the presence or absence of LPS (7 ng/ml) for 24 h. NO **(A)** and TNF-α **(B)** levels were measured in the media, and the cells were counted. Results are representatives of three independent experiments and are presented as mean ± SEM, *n* = 3–6; statistical significance was assessed by one-way analysis of variance followed by a Tukey–Kramer Multiple Comparison Test; ***P < 0.001 versus control.

The production of NO (Figure 2A) and TNF-α (Figure 2B) in non-stimulated BV2 cells or in BV2 cells induced by LPS (7 ng/ml) and treated with R-715 (10^−7^ and 10^−6^ M) was next considered. LPS markedly enhanced NO and TNF-α production, as compared with controls. R-715 at concentrations of 10^−7^ and 10^−6^ M significantly increased LPS-induced NO secretion (Figure 2A). R-715, at concentrations of 10^−7^ and 10^−6^ M, also increased LPS-induced TNF-α secretion (Figure 2B). By contrast, R-715 did not alter NO (Figure 2A) or TNF-α (Figure 2B) production in non-stimulated cells. However, the selective B_2_R antagonist HOE 140 failed to alter NO (Figure 3A) or TNF-α (Figure 3B) release from either non-stimulated or LPS-stimulated cells.

**Figure 4:**
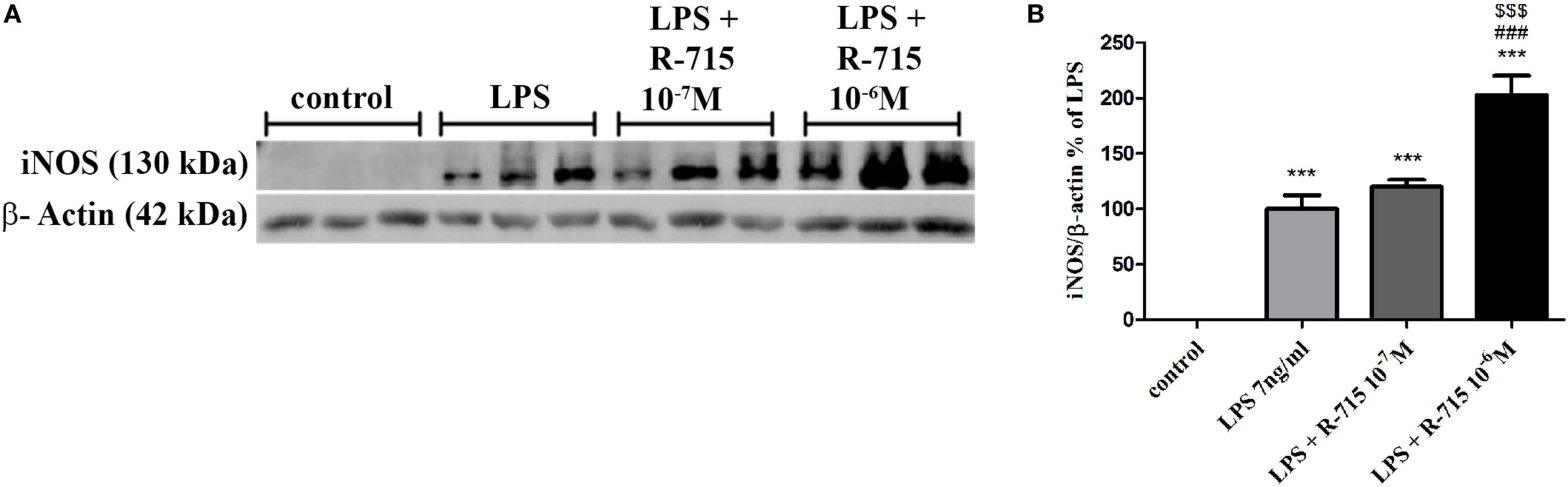
**Effect of the bradykinin B_1_ receptor (B_1_R) antagonist on iNOS expression in lipopolysaccharide (LPS)-stimulated BV2 cells**. Cells were incubated with the B_1_R antagonist R-715 (10^−7^ and 10^−6^ M) and LPS (7 ng/ml) for 24 h. Whole cell lysates (40 µg) were examined by Western blot analysis using anti-iNOS or β-actin antibodies. The results shown here represent three independent experiments **(A)**. The graph represents the mean ± SEM of three experiments **(B)**, *n* = 3. One-way analysis of variance and a Tukey–Kramer Test were used to determine statistical significance; ***P < 0.001 versus the control, ^###^*P* < 0.001 versus LPS, and ^$$$^*P* < 0.001 versus LPS + R-715 (10^−7^ M).

As shown in Figure 4, a 24-h treatment of BV2 cells with LPS (7 ng/ml) significantly increased iNOS expression levels. 10^−6^ M of R-715 increased the LPS-induced iNOS expression by up to 102%.

**Figure 5:**
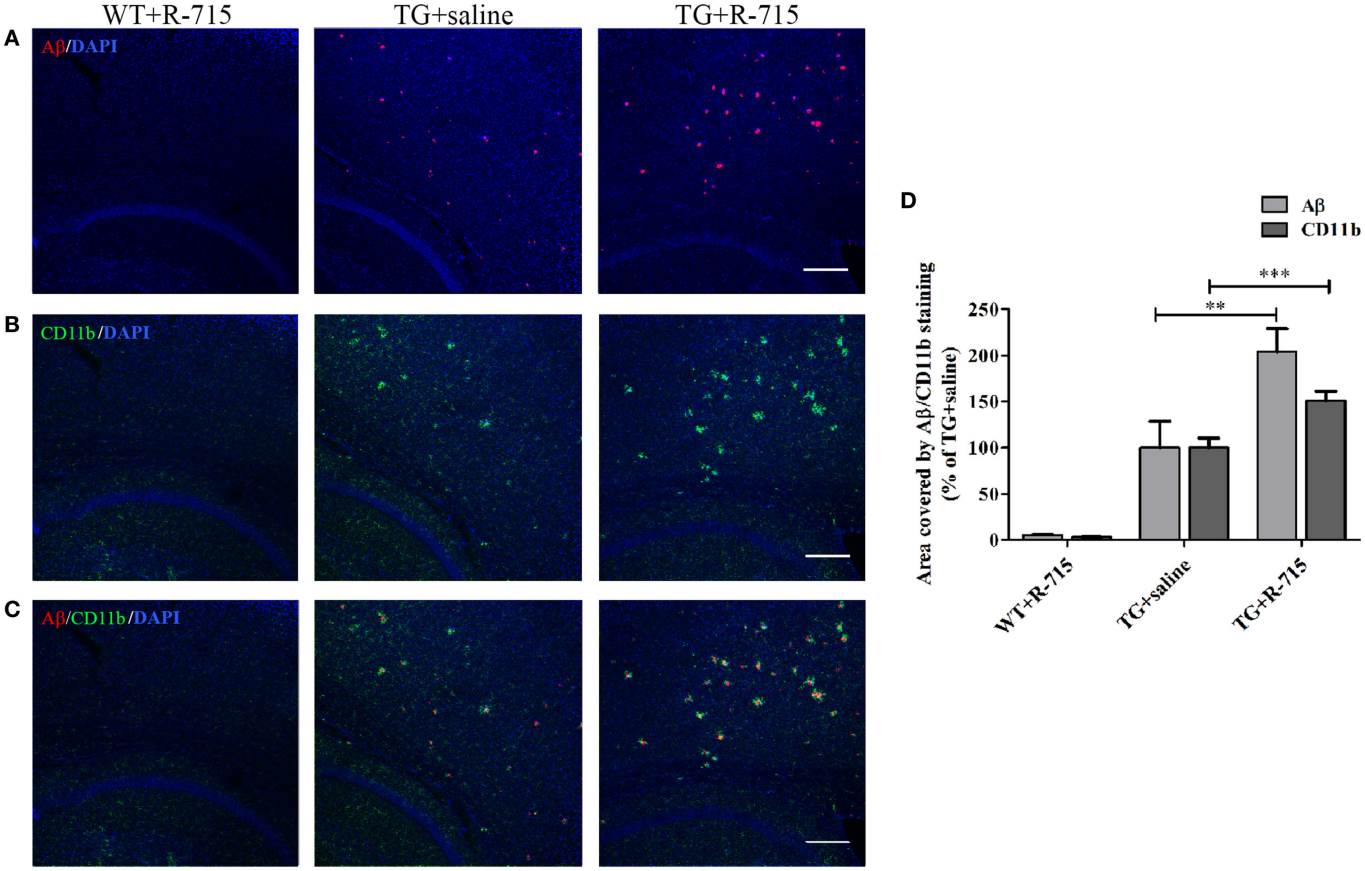
**Effects of intranasal delivery of the bradykinin B_1_ receptor antagonist on amyloid burden and the accumulation of microglial cells in the cortex of 5X familial AD (5XFAD) mice**. Two-month-old mice were treated with R-715 or the vehicle for 3½ weeks. After cardiac perfusion, their brains were cut into sections, stained for Aβ (*red*) **(A)** and CD11b (microglial accumulation) (*green*) **(B)** using appropriate antibodies and counter-stained with DAPI (*blue*). Representative confocal images of cortical sections from wild-type (WT) or 5XFAD mice (TG) are shown. **(C)** Merged images of Aβ and CD11b staining. The dose of intranasally administered R-715 was 1 mg/kg/day. Each group included 4–8 mice (*n* = 14–23 per experiment). The stained areas were quantified. The mean ± SEM of percentage of the stained areas is shown in the graphs, *n* ≥ 3 determinants **(D)**. For statistical comparisons, a one-way analysis of variance and Tukey–Kramer Multiple Comparison Test were conducted. ***P < 0.001 versus TG + saline; **P < 0.01 versus TG + saline. The scale bar is 200 µm.

**Figure 6:**
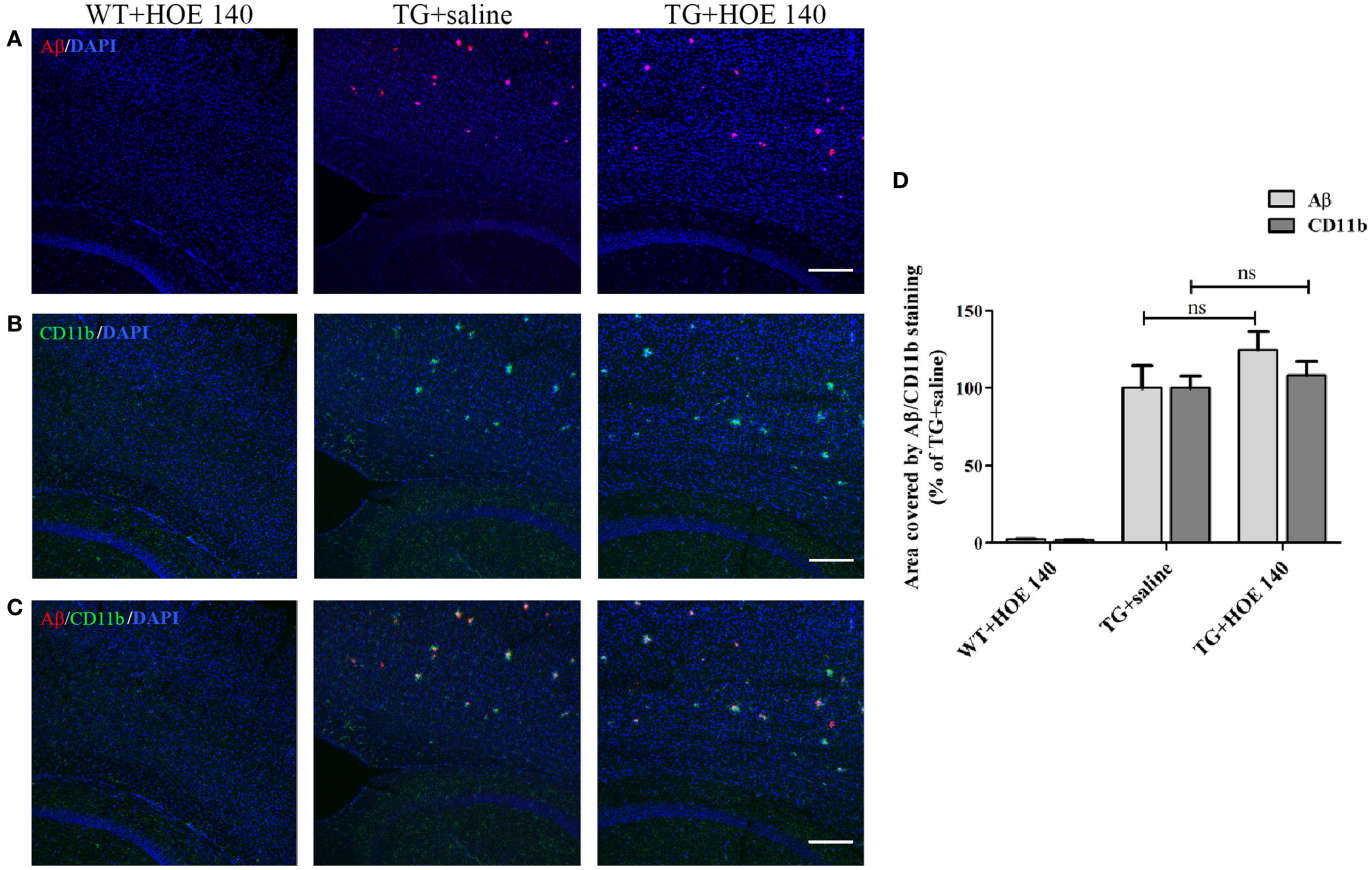
**The effects of intranasal delivery of the bradykinin B_2_ receptor antagonist on amyloid burden and the accumulation of microglial cells in the cortex of 5X familial AD (5XFAD) mice**. Two-month-old mice were treated with HOE 140 or the vehicle for 3½ weeks. After cardiac perfusion, their brains were cut into sections, stained for Aβ (*red*) **(A)** and CD11b (*green*) **(B)** using appropriate antibodies and counter-stained with DAPI (*blue*). Representative confocal images of cortical sections from wild-type (WT) or 5XFAD mice (TG) are shown. **(C)** Merged images of Aβ and CD11b staining. The dose of intranasally administered HOE 140 was 1 mg/kg/day. Each group included 4–7 mice (*n* = 15–19 per experiment). The stained areas were quantified. The mean ± SEM of percentage of the stained areas is shown in the graphs, *n* ≥ 3 determinants **(D)**. For statistical comparisons, a one-way analysis of variance and Tukey–Kramer Multiple Comparison Test were conducted. ns, non-significant. The scale bar is 200 µm.

As anticipated, the cortex of WT mice intranasally administered with R-715 did not show any Aβ plaques (Figure 5A). Levels of both Aβ and CD11b (marker for microglial accumulation) were significantly enhanced in age-matched 5XFAD mice treated intranasally with the vehicle (Figure 5). However, as compared to 5XFAD mice treated with the vehicle, age-matched 5XFAD mice treated intranasally with R-715 (1 mg/kg/day) showed close to 100 and 50% increases in Aβ burden (Figures 5A,D) and CD11b staining (Figures 5B,D), respectively. 5XFAD mice intranasally treated with HOE 140 did not display any differences in plaque burden (Figures 6A,D) or CD11b staining (Figures 6B,D) in the cortex, as compared to vehicle-treated mice. Modified Figures 5 and 6 are adapted with permission from Asraf et al. (28).

## Discussion

Although BK is generally viewed as a central pro-inflammatory mediator (25, 29, 30), a possible protective role for BK in the brain has been proposed. Recently, we showed a dual effect of kinins on the production of prostaglandin (PG), a pro-inflammatory mediator, in cultured glial cells (i.e., microglia and astrocytes) (31). Specifically, the activation of B_2_ receptors increased PG synthesis, whereas B_1_R agonists inhibited synthesis of these pro-inflammatory mediators. We have, therefore, suggested that a regulatory loop exists in which B_2_ receptors mediate enhanced-glial inflammation. B_1_ receptors are subsequently upregulated and are involved in the attenuation of glial inflammation (31). These findings were confirmed in part by Noda et al., who documented reduced microglial inflammation upon BK treatment of LPS-induced cells (32, 33). More recently, we also showed that a B_1_ receptor agonist abrogated NO and TNF-α production in LPS-treated BV2 microglial cells (26).

In the present study, R-715 significantly enhanced iNOS expression and release of TNF-α and NO from BV2 microglia. Using immunocytochemistry, we previously demonstrated the expression of both B_1_ and B_2_ receptor sub-types in BV2 microglia (34). In the present study, B_1_Rs are blocked by R-715, and no regulatory feedback inhibition exists through B_1_ receptors. This suggests that endogenous BK, possibly released from BV2 cells, activates B_2_ receptors and contributes to the amplification of inflammation (TNF-α and NO synthesis) induced by LPS. Interestingly, BK and LPS were shown to induce B_2_R expression and synergistically enhance nitrosative stress and inflammation in epithelial cells (35). Induction of activated microglial TNF-α and NO release is of particular relevance, as these pro-inflammatory cytokines are both associated with neuronal loss. iNOS expression and NO generation have been described in several brain pathologies, including AD (36). Microglial iNOS is induced by Aβ both *in vitro* and *in vivo* and activated iNOS-expressing microglia were found in amyloid plaques surrounded by dead and dystrophic neurons. Various modes and mechanisms by which NO can lead to neuronal death have been described (37, 38). TNF-α is also associated with neurodegeneration and furthermore induces the expression of amyloid precursor protein and promotes its cleavage by stimulating secretase activity to release Aβ. Reciprocally, Aβ induces TNF-α synthesis in neurons and glial cells. In addition, Aβ has been shown to physically bind TNFR-1, thereby inducing neuronal death (39).

For *in vivo* studies, the five familial Alzheimer’s disease (5XFAD) mouse model was employed. These mice develop rapid Aβ deposits alongside microglial accumulation beginning at 2 months of age, with plaques initially accumulate in the cortex. As shown in Figure 5, intranasal treatment of these mice with R-715 for 3.5 weeks significantly enhanced amyloid burden and CD11b expression in the cortex. Intranasal application of this B_1_R antagonist was chosen since this mode of delivery likely increases the direct action of the compound by bypassing the blood–brain barrier. Moreover, clinical studies involving nasal application of other compounds, such as insulin, to AD patients showed improvement in memory skills (40). However, peripheral effects of the antagonist cannot be ruled out. Furthermore, the effectiveness of the passage of peptides from nose to brain is controversial (41). Intranasal delivery of B_1_R or B_2_R antagonists has been tried here, for the first time. Similar results as those reported here were, nonetheless, reported by Passos et al., who showed that an 8 month-long treatment of triple mutant APP (Tg-SwDI) mice with R-715 resulted in enhanced amyloid burden (42). Antagonism of B_1_R using R-715 also resulted in significantly greater severity of multiple sclerosis in a mouse model of the disease (43). On the other hand, there is evidence suggesting a role for B_2_Rs in regulating brain inflammation and AD. Upregulation of B_1_ and B_2_ receptors in Aβ-infused rats was observed, mainly in brain regions such as the hippocampus and cortex, suggesting the possible involvement of kinins in AD (44). In a mouse model of AD, Prediger et al. showed improvement of cognitive deficits by genetic deletion or pharmacological antagonism of B_1_ or B_2_ receptors (45). Blockage of B_2_ receptor, as shown by Bicca et al., prevented Aβ-induced cognitive impairment by inhibiting brain inflammation (46). Moreover, differential roles of B_1_ and B_2_ receptors in memory consolidation were observed during aging in mice.

B_1_ and B_2_ receptors transduce their signals through similar cellular pathways. They both are generally described as signaling through Gαq and Gαi. However, B_2_ receptors also interact with other G proteins as well, including Gαs and Gα12/13. The signaling patterns are also different for both receptors. For example, in vascular smooth muscle cells, activating B_2_R induced transient increase in intracellular Ca^2+^ signaling, whereas B_1_ receptor stimulation was sustained (47). The specific way of signaling is possibly the result of different extent of regulation that these receptors are dependent on. Further studies are required to find out whether any of these differences explains differential involvement of BK receptors in modulation of amyloid burden and glial accumulation *in vitro* and *in vivo*.

To achieve better insight into mechanisms by which amyloid deposition is modulated by B_1_R, the effect of B_1_R antagonism on microglial/macrophage accumulation was investigated. R-715 distinctly augmented microglial/macrophage accumulation in the cortex of 5XFAD mice. Lee et al., similar to us, showed that reduced microglial activation was associated with less amyloid accumulation (48). Specific characterization of the microglia/macrophage phenotype(s) was not done here, although one can envision that a more complete analysis of microglial markers might point to a given functional or activation state that is more favorable for reducing amyloid accumulation.

To conclude, we have presented evidence supporting a role for B_1_R in brain inflammation and in the regulation of amyloid deposition in AD mice, possibly with microglial/macrophage involvement. Further studies are required to test whether modulation of this receptor can serve as a novel therapeutic strategy for AD.

## Ethics Statement

This study was carried out in accordance with the recommendations of the Institutional Animal Care and Use Committee of Ben-Gurion University of the Negev, Beer-Sheva, Israel. The protocol was approved by this committee: approval number IL-30-08-2011.

## Author Contributions

SF-B and KA designed the experiments. KA performed the experiments. KA and NT analyzed data and prepared figures. SF-B and KA wrote the manuscript.

## Conflict of Interest Statement

The authors state that the study was conducted without any commercial or financial relationships that could be construed as a potential conflict of interest. The reviewer, M-CT, and handling editor declared their shared affiliation, and the handling editor states that the process nevertheless met the standards of a fair and objective review.

## References

[1] Hebert LE Weuve J Scherr PA Evans DA. Alzheimer disease in the United States (2010-2050) estimated using the 2010 census. Neurology (2013) 80(19):1778–83. 10.1212/WNL.0b013e31828726f5 23390181 PMC3719424

[2] Hardy J Selkoe DJ. The amyloid hypothesis of Alzheimer’s disease: progress and problems on the road to therapeutics. Science (2002) 297(5580):353–6. 10.1126/science.1072994 12130773

[3] Holtzman DM Morris JC Goate AM. Alzheimer’s disease: the challenge of the second century. Sci Transl Med (2011) 3(77):77sr1. 10.1126/scitranslmed.3002369 21471435 PMC3130546

[4] Akiyama H Barger S Barnum S Bradt B Bauer J Cole GM Inflammation and Alzheimer’s disease. Neurobiol Aging (2000) 21(3):383–421. 10.1016/S0197-4580(00)00124-X 10858586 PMC3887148

[5] Heneka MT Carson MJ El Khoury J Landreth GE Brosseron F Feinstein DL Neuroinflammation in Alzheimer’s disease. Lancet Neurol (2015) 14(4):388–405. 10.1016/S1474-4422(15)70016-5 25792098 PMC5909703

[6] Kettenmann H Hanisch UK Noda M Verkhratsky A. Physiology of microglia. Physiol Rev (2011) 91(2):461–553. 10.1152/physrev.00011.2010 21527731

[7] Ji K Akgul G Wollmuth LP Tsirka SE. Microglia actively regulate the number of functional synapses. PLoS One (2013) 8(2):e56293. 10.1371/journal.pone.0056293 23393609 PMC3564799

[8] Parkhurst CN Yang G Ninan I Savas JN Yates JR III Lafaille JJ Microglia promote learning-dependent synapse formation through brain-derived neurotrophic factor. Cell (2013) 155(7):1596–609. 10.1016/j.cell.2013.11.030 24360280 PMC4033691

[9] Bamberger ME Harris ME McDonald DR Husemann J Landreth GE. A cell surface receptor complex for fibrillar beta-amyloid mediates microglial activation. J Neurosci (2003) 23(7):2665–74. 12684452 10.1523/JNEUROSCI.23-07-02665.2003 PMC6742111

[10] Paresce DM Ghosh RN Maxfield FR. Microglial cells internalize aggregates of the Alzheimer’s disease amyloid beta-protein via a scavenger receptor. Neuron (1996) 17(3):553–65. 10.1016/S0896-6273(00)80187-7 8816718

[11] Stewart CR Stuart LM Wilkinson K van Gils JM Deng J Halle A CD36 ligands promote sterile inflammation through assembly of a toll-like receptor 4 and 6 heterodimer. Nat Immunol (2010) 11(2):155–61. 10.1038/ni.1836 20037584 PMC2809046

[12] Liu Y Walter S Stagi M Cherny D Letiembre M Schulz-Schaeffer W LPS receptor (CD14): a receptor for phagocytosis of Alzheimer’s amyloid peptide. Brain (2005) 128(Pt 8):1778–89. 10.1093/brain/awh531 15857927

[13] von Bernhardi R Ramirez G Toro R Eugenin J. Pro-inflammatory conditions promote neuronal damage mediated by amyloid precursor protein and decrease its phagocytosis and degradation by microglial cells in culture. Neurobiol Dis (2007) 26(1):153–64. 10.1016/j.nbd.2006.12.006 17240154

[14] Noda M Kariura Y Amano T Manago Y Nishikawa K Aoki S Expression and function of bradykinin receptors in microglia. Life Sci (2003) 72(14):1573–81. 10.1016/S0024-3205(02)02449-9 12551746

[15] Ifuku M Farber K Okuno Y Yamakawa Y Miyamoto T Nolte C Bradykinin-induced microglial migration mediated by B1-bradykinin receptors depends on Ca2+ influx via reverse-mode activity of the Na+/Ca2+ exchanger. J Neurosci (2007) 27(48):13065–73. 10.1523/JNEUROSCI.3467-07.2007 18045900 PMC6673405

[16] Bhoola KD Figueroa CD Worthy K Bioregulation of kinins: kallikreins, kininogens, and kininases. Pharmacol Rev (1992) 44(1):1–80. 1313585

[17] Lai J Luo MC Chen Q Porreca F. Pronociceptive actions of dynorphin via bradykinin receptors. Neurosci Lett (2008) 437(3):175–9. 10.1016/j.neulet.2008.03.088 18450375 PMC2767248

[18] Cruden NL Newby DE. Therapeutic potential of icatibant (HOE-140, JE-049). Expert Opin Pharmacother (2008) 9(13):2383–90. 10.1517/14656566.9.13.2383 18710362

[19] Abraham WM Scuri M Farmer SG. Peptide and non-peptide bradykinin receptor antagonists: role in allergic airway disease. Eur J Pharmacol (2006) 533(1–3):215–21. 10.1016/j.ejphar.2005.12.071 16455073

[20] Walker K Perkins M Dray A Kinins and kinin receptors in the nervous system. Neurochem Int (1995) 26(1):1–16. 10.1016/0197-0186(94)00114-A 7787759

[21] Kamiya T Katayama Y Kashiwagi F Terashi A. The role of bradykinin in mediating ischemic brain edema in rats. Stroke (1993) 24(4):571–5. 10.1161/01.STR.24.4.571 7682018

[22] Iores-Marcal LM Viel TA Buck HS Nunes VA Gozzo AJ Cruz-Silva I Bradykinin release and inactivation in brain of rats submitted to an experimental model of Alzheimer’s disease. Peptides (2006) 27(12):3363–9. 10.1016/j.peptides.2006.08.012 17030465

[23] Regoli D Barabe J Pharmacology of bradykinin and related kinins. Pharmacol Rev (1980) 32(1):1–46. 7015371

[24] Calixto JB Cabrini DA Ferreira J Campos MM Kinins in pain and inflammation. Pain (2000) 87(1):1–5. 10.1016/S0304-3959(00)00335-3 10863040

[25] Gimpl G Walz W Ohlemeyer C Kettenmann H. Bradykinin receptors in cultured astrocytes from neonatal rat brain are linked to physiological responses. Neurosci Lett (1992) 144(1–2):139–42. 10.1016/0304-3940(92)90735-P 1331908

[26] Sarit BS Lajos G Abraham D Ron A Sigal FB Inhibitory role of kinins on microglial nitric oxide and tumor necrosis factor-alpha production. Peptides (2012) 35(2):172–81. 10.1016/j.peptides.2012.03.026 22490447

[27] Oakley H Cole SL Logan S Maus E Shao P Craft J Intraneuronal beta-amyloid aggregates, neurodegeneration, and neuron loss in transgenic mice with five familial Alzheimer’s disease mutations: potential factors in amyloid plaque formation. J Neurosci (2006) 26(40):10129–40. 10.1523/JNEUROSCI.1202-06.2006 17021169 PMC6674618

[28] Asraf K Torika N Roasso E Fleisher-Berkovich S. Differential effect of intranasally administrated kinin B1 and B2 receptor antagonists in Alzheimer’s disease mice. Biol Chem (2016) 397(4):345–51. 10.1515/hsz-2015-0219 26556847

[29] Austinat M Braeuninger S Pesquero JB Brede M Bader M Stoll G Blockade of bradykinin receptor B1 but not bradykinin receptor B2 provides protection from cerebral infarction and brain edema. Stroke (2009) 40(1):285–93. 10.1161/STROKEAHA.108.526673 18988906

[30] Couture R Harrisson M Vianna RM Cloutier F Kinin receptors in pain and inflammation. Eur J Pharmacol (2001) 429(1–3):161–76. 10.1016/S0014-2999(01)01318-8 11698039

[31] Levant A Levy E Argaman M Fleisher-Berkovich S. Kinins and neuroinflammation: dual effect on prostaglandin synthesis. Eur J Pharmacol (2006) 546(1–3):197–200. 10.1016/j.ejphar.2006.06.074 16889769

[32] Noda M Kariura Y Pannasch U Nishikawa K Wang L Seike T Neuroprotective role of bradykinin because of the attenuation of pro-inflammatory cytokine release from activated microglia. J Neurochem (2007) 101(2):397–410. 10.1111/j.1471-4159.2006.04339.x 17402969

[33] Noda M Sasaki K Ifuku M Wada K. Multifunctional effects of bradykinin on glial cells in relation to potential anti-inflammatory effects. Neurochem Int (2007) 51(2–4):185–91. 10.1016/j.neuint.2007.06.017 17669557

[34] Fleisher-Berkovich S Filipovich-Rimon T Ben-Shmuel S Hulsmann C Kummer MP Heneka MT Distinct modulation of microglial amyloid beta phagocytosis and migration by neuropeptides (i). J Neuroinflammation (2010) 7:61 10.1186/1742-2094-7-61 20937084 PMC2964654

[35] Ricciardolo FL Sorbello V Benedetto S Defilippi I Sabatini F Robotti A Bradykinin- and lipopolysaccharide-induced bradykinin B2 receptor expression, interleukin 8 release and “nitrosative stress” in bronchial epithelial cells BEAS-2B: role for neutrophils. Eur J Pharmacol (2012) 694(1–3):30–8. 10.1016/j.ejphar.2012.07.051 22935637

[36] Brown GC. Mechanisms of inflammatory neurodegeneration: iNOS and NADPH oxidase. Biochem Soc Trans (2007) 35(Pt 5):1119–21. 10.1042/BST0351119 17956292

[37] Gibbons HM Dragunow M. Microglia induce neural cell death via a proximity-dependent mechanism involving nitric oxide. Brain Res (2006) 21(1):1–15. 10.1016/j.brainres.2006.02.032 16564033

[38] Diaz A Mendieta L Zenteno E Guevara J Limon ID. The role of NOS in the impairment of spatial memory and damaged neurons in rats injected with amyloid beta 25-35 into the temporal cortex. Pharmacol Biochem Behav (2011) 98(1):67–75. 10.1016/j.pbb.2010.12.005 21163295

[39] Wang WY Tan MS Yu JT Tan L Role of pro-inflammatory cytokines released from microglia in Alzheimer’s disease. Ann Transl Med (2015) 3(10):136 10.3978/j.issn.2305-5839.2015.03.49 26207229 PMC4486922

[40] Freiherr J Hallschmid M Frey WH II Brunner YF Chapman CD Holscher C Intranasal insulin as a treatment for Alzheimer’s disease: a review of basic research and clinical evidence. CNS Drugs (2013) 27(7):505–14. 10.1007/s40263-013-0076-8 23719722 PMC3709085

[41] Leng G Ludwig M. Intranasal oxytocin: myths and delusions. Biol Psychiatry (2016) 79(3):243–50. 10.1016/j.biopsych.2015.05.003 26049207

[42] Passos GF Medeiros R Cheng D Vasilevko V Laferla FM Cribbs DH The bradykinin B1 receptor regulates Abeta deposition and neuroinflammation in Tg-SwDI mice. Am J Pathol (2013) 182(5):1740–9. 10.1016/j.ajpath.2013.01.021 23470163 PMC3644719

[43] Schulze-Topphoff U Prat A Prozorovski T Siffrin V Paterka M Herz J Activation of kinin receptor B1 limits encephalitogenic T lymphocyte recruitment to the central nervous system. Nat Med (2009) 15(7):788–93. 10.1038/nm.1980 19561616 PMC4903020

[44] Viel TA Lima Caetano A Nasello AG Lancelotti CL Nunes VA Araujo MS Increases of kinin B1 and B2 receptors binding sites after brain infusion of amyloid-beta 1-40 peptide in rats. Neurobiol Aging (2008) 29(12):1805–14. 10.1016/j.neurobiolaging.2007.04.019 17570564

[45] Prediger RD Medeiros R Pandolfo P Duarte FS Passos GF Pesquero JB Genetic deletion or antagonism of kinin B(1) and B(2) receptors improves cognitive deficits in a mouse model of Alzheimer’s disease. Neuroscience (2008) 151(3):631–43. 10.1016/j.neuroscience.2007.11.009 18191900

[46] Bicca MA Costa R Loch-Neckel G Figueiredo CP Medeiros R Calixto JB B(2) receptor blockage prevents Abeta-induced cognitive impairment by neuroinflammation inhibition. Behav Brain Res (2015) 278:482–91. 10.1016/j.bbr.2014.10.040 25446751

[47] Mathis SA Criscimagna NL Leeb-Lundberg LM. B1 and B2 kinin receptors mediate distinct patterns of intracellular Ca2+ signaling in single cultured vascular smooth muscle cells. Mol Pharmacol (1996) 50(1):128–39. 8700105

[48] Lee DC Rizer J Hunt JB Selenica ML Gordon MN Morgan D. Review: experimental manipulations of microglia in mouse models of Alzheimer’s pathology: activation reduces amyloid but hastens tau pathology. Neuropathol Appl Neurobiol (2013) 39(1):69–85. 10.1111/nan.12002 23171029 PMC4300851

